# Procedures to Develop a Computerized Adaptive Testing to Advance the Measurement of Narcissistic Personality

**DOI:** 10.3389/fpsyg.2020.01437

**Published:** 2020-06-30

**Authors:** Hao Luo, Yan Cai, Dongbo Tu

**Affiliations:** School of Psychology, Jiangxi Normal University, Nanchang, China

**Keywords:** computerized adaptive testing, *DSM-5*, item response theory, measurement, narcissistic personality

## Abstract

Narcissistic personality (NP) has recently attracted a great deal of attention. In this study, we mainly investigated the feasibility and effectiveness of using computerized adaptive testing (CAT) to measure NP (CAT-NP). The CAT in this study was simulated by the responses of several NP questionnaires of 1,013 university students as if their responses were collected adaptively. The item bank (85 items) that met the requirements of the psychometric properties of Item Response Theory (IRT) was first established, and then the CAT dynamically selected items according to the estimates of current trait level until the prespecified measurement precision is achieved. Finally, the efficiency and validity of the CAT were verified. The results showed that the proposed CAT-NP had reasonable reliability, validity, predictive utility, and high correlation. In addition, the CAT-NP could significantly save item usage without losing measurement accuracy, which greatly improves the test efficiency. The advantages and limitations of CAT in measuring NP and other psychological tests are discussed in the final section.

## Introduction

Narcissism, as a concept of psychology, can be traced back to Narcissus of ancient Greek mythology (Frederic et al., 2010). The concept was first presented by Havelock Ellis and early psychoanalytic theorists, and then Freud was the first to systematically discuss the issue of narcissism (Crockatt, 2006). So far, narcissism still has no unified and strict operational definition. At the clinical level, narcissism is seen as a personality disorder, so narcissistic personality (NP) disorder cannot be measured by questionnaire alone. This article does not discuss narcissism from a clinical perspective but focuses on the characteristics of NP that are common in normal groups. The NP studied in this article can cause problems in many aspects of life, such as relationships, work, school, and finances (Ab Saleh and Awada, 2016). Persons with NP usually feel unhappy and disappointed if they do not get the special help or admiration they think they deserve. Research has shown that that modern life makes us more narcissistic (Twenge et al., 2014). A notable research direction is to discover rapid measurement of these high-risk populations.

However, current efforts to measure NP are inadequate. The first problem is the representativeness of the measuring tools. Until the American Psychiatric Association (APA) developed the *Diagnostic and Statistical Manual of Mental Disorders* (5th edition; *DSM-5*; American Psychiatric Association, 2013), there had not been a relatively uniform standard previously until DSM-5. Since then, more and more measuring tools have been developed based on this criteria, such as the Structural Clinical Interview for *DSM-IV* Axis II disorders Patient Questionnaire (SCID-II; First et al., 1995), Personality Diagnostic Questionnaire-Fourth Edition (PDQ-4; Hyler, 1994), Narcissistic Personality Questionnaire (NPQ; Motter, 2009), etc. Research shows that PDQ-4 and SCID-II are not widely applicable for measuring NP because they have few items (Miller and Campbell, 2008). In comparison, NPQ and FFNI are more popular and complete as NP measuring tools (Motter, 2009). Another problem is the use of measurement theory. In previous studies, the scales used to measure NP were based on the Classical Test Theory (CTT) framework, while these scales have fixed lengths and usually contain items corresponding to various levels of NP. For example, the NPQ and the FFNI are often used for measuring NP (Motter, 2009). However, these two questionnaires have a large number of items, 73 and 145 respectively. Many items may deviate from the symptoms of respondents with NP, so that all respondents are commonly required to answer all items of a questionnaire, which may increase the respondents’ unnecessary measurement burden and then reduce respondents’ enthusiasm as well as the measurement accuracy. It is very important to not only provide a whole measure of NP but also to reduce the test burden and improve the precision of measurement. Therefore, the focus of our attention is to discover how to make rational use of these questionnaires for quick and efficient measurement of NP.

With the development of modern educational and psychological measurement theory and computer technology, computerized adaptive testing (CAT) has emerged. Different from the traditional paper-and-pen test, CAT can effectively reduce the test length while achieving measurement accuracy, which greatly reduces the test burden (Drasgow and Olson-Buchanan, 1999). Another attractive feature of CAT is that it is available to match a set of the most suitable items for each participant with a different trait level. In this way, it can make the test more intelligent, reduce the test length or test time, and estimate the psychological trait level of the subjects effectively and accurately (Pilkonis et al., 2014). Initially, the CAT was designed for cognitive testing (Wainer et al., 2001). More recently, various CAT procedures for general mental health assessments have been developed (Reise and Henson, 2000), such as CAT-ANX for measuring anxiety (Gibbons et al., 2014), D-CAT for measuring depression (Fliege et al., 2005), CAT-Integrity for measuring integrity (Egberink and Veldkamp, 2007), etc. The emergence of these CAT versions shows the universality and effectiveness of CAT technology in psychological testing. In addition, some CAT versions cater to personality traits, such as CAT-SPQ (Moore et al., 2018) and the CAT-PD project (Simms et al., 2011); the former uses only the 74-item Schizotypal Personality Questionnaire (SPQ; Raine, 1991) as the item bank. Research has shown that the measurement results of the 16-item CAT and 74-item full SPQ are close, and the classification accuracy of the 16-item CAT is better than that of the 22-item SPQ-Brief (Moore et al., 2018). This shows that CAT technology has high efficiency in measuring personality traits and it is very important to establish CAT in measuring personality traits. While the CAT-PD project is a measure of the whole personality traits by the International Personality Item Pool (IPIP; Goldberg et al., 2006). On the one hand, the established CAT item bank allows the scales to be directly compared, because the item parameters in the item bank are on the same scale. On the other hand, the item bank can basically cover the measurement of the entire population, and the measurement reliability of each person is basically consistent. Last but not least, CAT can greatly reduce the testing burden of the subjects.

To the best of our knowledge, the CAT measurement for NP has not been formally discussed in the literature. Therefore, the method, algorithm, and implementation of CAT to measure NP are worth researching. The purpose of this article is to construct the item bank of CAT for measuring NP (CAT-NP) and then investigate the efficiency, reliability, and validity of CAT-NP.

## Materials and Methods

### Participants

A total of 1,013 undergraduates from five universities in China were recruited for this study. The average age of these participants was 21.35 years (*SD* = 1.84, range 18–26) and females (65.7%) made up most of this sample. The detailed demographic information is shown in Table 1. Moreover, participants voluntarily participated in the survey, and written informed consent was obtained in accordance with the Declaration of Helsinki.

**TABLE 1:** Demographic characteristics (*N* = 1,013).

**Variables**	**Category**	**Frequency**	**Percent (%)**
Gender	Male	347	34.3
	Female	666	65.7
Age	18–20	325	32.1
	21–23	540	53.3
	24–26	108	10.7
	Missing	40	3.9
Region	City	483	47.7
	Rural	530	52.3
Whether the only child or not	Yes	302	29.8
	No	703	69.4
	Missing	8	0.8

### Measures

We found that there are not many scales that specifically measure NP. These questionnaires include a 16-item Narcissistic Personality Inventory (NPI-16; Ames et al., 2006), the Narcissistic Personality Questionnaire (NPQ; Motter, 2009), the Five-Factor Narcissism Inventory (FFNI; Glover et al., 2012), and the Personality Diagnostic Questionnaire (PDQ-4; Hyler, 1994). It is worth noting that the PDQ-4 is not in the item bank because the PDQ-4 is used as a criterion questionnaire in this study. More specifically, the Chinese version of PDQ-4 used in this article was developed by Yang et al. (2000). The other three scales (NPI-16, NPQ, FFNI) were first translated into Chinese by four assessors, including two experts in English and two experts in psychology/psychometrics. Their task was to judge the accuracy of translation and relevance/suitability of each item for measuring NP in the sociocultural context of China. Then the study applied 30 Chinese college students to verify the quality and accuracy of the second draft by cognitive interview. Finally, the Chinese version of the three scales (NPI-16, NPQ, FFNI) was formed.

#### 16-Item Narcissistic Personality Inventory (NPI-16)

The 16-item Narcissistic Personality Inventory is a simplified version of the Narcissistic Personality Inventory by Ames et al. (2006). All the items measure a general construct of trait narcissism and the Cronbach’s alpha is 0.72 (Ames et al., 2006; Glover et al., 2012; Furnham et al., 2014). The Chinese version of NPI-16 has a Cronbach’s alpha of 0.64 in the current study.

#### Narcissistic Personality Questionnaire (NPQ)

The Narcissistic Personality Questionnaire was based on the 9 criteria for narcissism in *DSM-IV-TR* with 73 items (Motter, 2009). All the items are 0–1 scoring, and the Cronbach’s alpha is 0.88 (NPQ; Motter, 2009). In the current study, the Chinese version of NPQ has a Cronbach’s alpha of 0.89, and its correlation with the criterion questionnaire (PDQ-4) is 0.70. Compared with NPI, NPQ is a more mature tool for measuring narcissism (Motter, 2009).

#### The Five-Factor Narcissism Inventory (FFNI)

The Five-Factor Narcissism Inventory is a self-report measure of NP by Glover et al. (2012). This inventory contains 148 items, and each item is scored on a 1–5 scale. The FFNI involved the following 15 subscales: reactive anger, shame, indifference, need for admiration, exhibitionism, authoritativeness, grandiose fantasies, manipulativeness, exploitativeness, entitlement, lack of empathy, arrogance, acclaim-seeking, thrill-seeking, and distrust. These subscales correspond to the Five-Factor Model (FFM) of narcissism personality structure (Glover et al., 2012). The Cronbach’s alpha for this scale was 0.90, and the convergent validity of the FFNI with PDQ-4 was 0.68, which was significant at the 0.001 level (Glover et al., 2012). In the current study, the Chinese version of FFNI has a Cronbach’s alpha of 0.86, and its correlation with the criterion questionnaire (PDQ-4) is 0.57.

#### Personality Diagnostic Questionnaire–Fourth Edition (PDQ-4)

The PDQ-4 (Hyler, 1994) is a 99-item true–false self-report measure of personality psychopathology. The NP scale contains nine items, which are representative of the *DSM-IV* NP diagnostic criteria, and the Cronbach’s alpha was 0.78 (Glover et al., 2012). Research shows that the Cronbach’s alpha of the PDQ-4 in China is 0.64 (Yang et al., 2000). In the present study, the Cronbach’s alpha for this scale was 0.60.

### Construction of the Item Bank for CAT-NP

#### Unidimensionality

Unidimensionality is a necessary condition for the IRT-based CAT and it means that there is a potential trait variable that reflects common differences between items (Embretson and Reise, 2000). According to the literature, when a one-dimensional test accounts for at least 20% of the test variance (Reckase, 1979) and the ratio of explained variance of the first factor to the second factor is greater than 4, it can be considered that the scale conforms to the unidimensionality hypothesis (Flens et al., 2016). This study used the aforementioned criterion to investigate the unidimensionality of the item bank.

#### IRT Model Selection

The logistic model is a prevalent and basic IRT model, and the model is a regression of the response probability of the subjects to their potential trait levels. The IRT model consists of item parameters (e.g., discrimination and threshold parameters) and person latent trait parameters (θ). Because the threshold parameter (*b*) and the latent trait level parameters (θ) are defined on the same scale, for a subject whose θ is known, the probability of response can be predicted by the model; something that CTT cannot do. The item response function of the two-parameter logistic model is expressed as

(1) $$P\left(X_{i\,j}=1|\theta_{i},a_{j},b_{j}\right)=\frac{1}{1+\exp\left(-1.702\,a_{j}\,\left(\theta_{i}-b_{j}\right)\right)},$$

where *a*_*j*_ and *b*_*j*_ represent the discrimination parameter and difficulty/threshold parameter of item *j* respectively, θ_*i*_ represents the latent trait parameter of the subject *i*, and *P*(*X*_*ij*_ = 1|θ_*i*_,*a*_*j*_,*b*_*j*_) indicates the response probability of the subject *i* on item *j*. By the logistic model, different polytomous scored IRT models have been developed, such as the Graded Response Model (GRM; Samejima, 1969), which is a generalized version of the two-parameter logistic model. In the GRM, the probability of getting *t* points is defined as the probability of getting *t* points and above *t* points minus the probability of getting *t* + 1 points and above *t* + 1 points, as follows:

(2) $$P\left(X_{i\,j}=t\right)=P\left(X_{i\,j}\geq t\right)-P\left(X_{i\,j}\geq t+1\right)$$

Here

(3) $$P\left(X_{i\,j}\geq t\right)=\frac{1}{1+\exp\left[-1.702\,a_{i}\,\left(\theta_{i}-b_{j\,t}\right)\right]},$$

where *b*_*jt*_ is the difficulty/threshold parameter of item *j* on category *t*.

In the initial item bank, there are both dichotomous and polytomous items. In this study, we compare the goodness of fit of several IRT models with the real data and select the IRT model with the best goodness of fit via the Akaike information criterion (AIC; Akaike, 1974), Bayesian information criterion (BIC; Schwarz, 1978), and the -2 log likelihood (-2LL; Spiegelhalter et al., 1998). Those IRT models are the Graded Response Model (GRM; Samejima, 1969), the Generalized Partial Credit Model (GPCM; Muraki, 1997), and the Partial Credit Model (PCM; Masters and Wright, 1997), which can be used to analyze the data including the dichotomous and polytomous items. In general, we chose the IRT model with the smaller indices. Model selection was conducted by using the R package mirt (Version 1.24; Chalmers, 2012).

#### Local Independence

Local independence is a necessary assumption of IRT, which means that the responses of the subjects on each item are independent and not affected by the other items. In this study, the *Q*_3_ statistic (Yen, 1993) was used as the index to test local independence. According to the rules proposed by Cohen (1988), the deviation is in a reasonable range when *Q*_3_ is between 0.26 and 0.36; when the *Q*_3_ exceeds 0.36, it indicates that there is a large deviation between the items and means that the items are not independent. In this case, one item with a larger cumulative quantity of *Q*_3_ (*Q*_3_ > 0.36) should be deleted from the two items compared. Local independence analysis was conducted via the R package mirt (Version1.24; Chalmers, 2012).

#### Item Parameters

Items with a high discrimination parameter can distinguish different levels of subjects and measure latent traits more accurately. Therefore, in the CAT, item discrimination parameter is a very important index to measure the quality of the item. To estimate the trait level parameters of the subjects effectively, the acceptable range of item discrimination is 0.5–2.5 (Chang and Ying, 1996). To ensure that there are high-quality items in the item bank, this study excludes the items whose discrimination is less than 0.5. Item parameters analysis was conducted via the R package mirt (Version1.24; Chalmers, 2012).

#### Item Fit Testing

The item fit testing is used to check whether the item fits with the IRT model, and the *S-*χ*^2^* statistics (Kang and Chen, 2008) was used to carry out the item fit testing. When the *p*-value of *S-*χ*^2^* for an item is less than the threshold value (such as 0.05 or 0.01), the response probability predicted by the IRT model does not fit with the response probability of the actual data, and this item should be considered to be deleted (Orlando and Thissen, 2003). Item fit was also conducted by using the R package mirt (Version 1.24; Chalmers, 2012).

#### Differential Item Functioning

Differential item functioning (DIF) is used to determine systemic bias caused by demographic variables such as gender, age, region, etc. (Gaynes et al., 2002). In short, the DIF appears when two groups with the same latent traits have different responses on the item, to discern whether there is a measurement bias in the items having an important influence on the estimation of the trait level of the subjects. The CAT is always a shorter test than other tests so that DIF may have a more significant impact (Wainer et al., 2000). Therefore, the DIF test under the CAT is crucial. In this study, the logistic regression (LR) method was used to carry out the DIF test and used the method of McFadden’s pseudo *R*^2^ to evaluate (Crane et al., 2006). When the change of *R*^2^ was greater than 0.02 (Choi et al., 2011), it indicates that DIF exists in the item. DIF analysis was conducted using the R package lordif (Version 0.3-3; Choi, 2015).

### Simulation of the CAT-NP

The CAT simulation study with the real participants’ responses data in a paper-and-pencil test (P&P) was conducted to investigate the characteristics, marginal reliability, and correlation of the CAT-NP. The whole simulation program of CAT is simulated in R software. Typically, the CAT system includes five essential building blocks (Weiss and Kingsbury, 1984): calibrated item bank, starting level, item selection, scoring method, and stopping rule. The remaining four sections are described in the text that follows.

#### Starting Level

The CAT adaptively selects items according to the current responses of the subjects. Considering that the subjects have no response before answering the first item, CAT needs to give a starting level. There are three methods for the starting level. The first is the background information obtained through other channels, such as grades in school, IQ, and so on. The second method is to assume that the subjects have an intermediate level of latent trait and select an item of medium difficulty. The third is to randomly select the first item from the item bank. In this study, the first item given to the subjects was randomly selected from the item bank.

#### Item Selection

If the CAT has an estimate of the latent trait of the subjects, then it can select the item that is most suitable for that estimated value by an item selection index. The most commonly used item selection index is called the information function, where an item that makes this function larger means more information can be provided. In other words, the item provides the least measurement error. To reach the prespecified measurement precision quickly, the item with the most information will be chosen. Many information functions were developed from different domains. For example, the Fisher information function was proposed from statistics while the Shannon information function, Kubek–Leibler information function, mutual information function, and so on were developed from computer science. The maximum Fisher information criterion (MIC) was used here to select items after the first item in this study.

#### Scoring Method

After the subjects finish an item, CAT needs to update or estimate their trait level by a statistical method that is called scoring method. Three typical scoring methods mainly include the maximum likelihood estimation (MLE), maximum *a posteriori* estimation (MAP), and expected *a posteriori* (EAP) estimation. More precisely, MLE is different from the last two methods in that MLE is an unbiased estimate. When all the answers are either correct or wrong, the MLE method will fail, and the MAP or EAP is required. In this study, a relatively simple, efficient, and stable EAP method was applied to estimate the latent trait parameters of the subjects.

#### Stopping Rule

The stopping rule is generally based on whether the trait level has reached the prespecified accuracy. Item information is generally used to measure this precision in CAT. The fixed number of items administered is also a stopping rule, which means that all subjects finished the test when the test length has reached the prespecified length. Sometimes the two methods are used together. In IRT, the relationship between information (*I*), standard errors (*SE*) of measurement, and reliability (ρ) can be represented by the following formula when the mean and *SD* of theta (θ) are fixed to 0 and 1, respectively:

(4) $$\rho =1-S\,E^{2}=1-\frac{1}{I}.$$

Aiken (1997) summarized the data of previous studies and concluded that the values of low reliability, medium reliability, and high reliability in personality test were 0.46, 0.85, and 0.97 under CTT, respectively.

Based on the relationship between test reliability and test information, the stopping rule of reaching the prescribed reliability (ρ) was adopted to ensure the accuracy of latent trait estimation and the same measurement accuracy for each subject. To avoid some subjects completing all the items in the item bank because of failure to reach the prescribed reliability, the CAT set the maximum length of items at 35. Based on previous research, the minimum reliability was set to 0.90, 0.85, 0.80, 0.75, and 0.70 respectively to investigate the performance of CAT under different precision requirements.

### Characteristics of the CAT-NP

To investigate the efficiency of the proposed CAT, the correlation coefficient was obtained between the values of trait level estimated by CAT and the values of trait level estimated by the paper-and-pencil test of the whole item bank. At the same time, the average usage item length, measurement error, and measurement reliability under different stopping rules of the CAT were investigated. The lower the average used item length is, the higher is the efficiency of the test. The lower average standard error is, the higher is the accuracy of the test. Lastly, the greater the correlation coefficient is, the higher is the consistency between CAT test and the traditional test.

#### Criterion-Related Validity and Predictive Utility (Sensitivity and Specificity) of CAT-NP

To investigate the predictive utility of CAT, the 1,013 participants also completed the scale of PDQ-4-NP which is not included in the item bank. This article first compares the results of all participants on the CAT with those on the scale of PDQ-4-NP and judges the predictive accuracy of the CAT by calculating the correlation coefficient, sensitivity, and specificity. Here, we also introduce the area under curve (AUC), which is defined as the area below the receiver operating characteristic (ROC) curve. The larger the area under the curve is, the higher the measuring accuracy. According to Kraemer and Kupfer’s research, the closer the AUC is to 1, the better the measuring effect (Kraemer and Kupfer, 2006). The AUC is between 0.7 and 0.9, indicating that there is better accuracy, while it indicates that there is a high accuracy when AUC is greater than 0.9 (Forkmann et al., 2013). On the determination of the critical value, it was manifested generally by maximizing the Youden Index (YI = sensitivity + specificity - 1) (Schisterman et al., 2005). Additionally, in this study, sensitivity means the probability that a patient is accurately diagnosed with a disease, while specificity refers to the probability that a healthy person is diagnosed with no disease. These two indexes range from 0 to 1, and the larger they are, the better the measuring effect will be.

## Results

### Construction of Item Bank for CAT-NP

#### Unidimensional

The exploratory factor analysis (EFA) of all items in the initial item bank shows that some items have small loads on the first factor or large loads on two or more factors. After deleting these items (including 46 items), we reran EFA, and the results indicate that the first eigenvalue is 19.223, the second eigenvalue is 3.721, and their ratio is 5.166. Moreover, the variance explained by the first factor is 20.024%. The result satisfies the condition that the ratio of the first and the second eigenvalues is greater than 4 and the variance explained by the first eigenvalue is greater than 20% (Reckase, 1979). Therefore, according to the criterion of Reckase (1979), the remaining item bank (90 items) satisfies the unidimensional hypothesis of IRT.

#### IRT Model Selection

Table 2 documents the model-fit indices of three IRT models that include GRM, GPCM, and PCM. Results show that the GRM has smaller values of deviation, AIC, and BIC, which indicates that the GRM fits the item bank better than GPCM and PCM. Therefore, the GRM is selected to conduct the subsequent IRT analysis.

**TABLE 2:** Model-fit indices.

**Model**	**Deviation**	**AIC**	**BIC**
GRM	101,774.9	102,216.9	103,304.4
GPCM	101,834.9	102,276.9	103,364.4
PCM	104,012.8	104,286.8	104,960.9

*AIC, Akaike’s information criterion; BIC, Bayesian information criterion; Deviation, -2log-likelihood; GPCM, Generalized Partial Credit Model; GRM, Graded Response Model; PCM, Partial Credit Model.*

#### Local Independence

According to the criterion that *Q*_3_ needs to be less than 0.36, we find that two items do not conform to the local independence hypothesis and two items were deleted. One pair of items is “Sometimes I daydream about being famous” and “I often fantasize about someday being famous,” respectively. There were 88 remaining items in the item bank after excluding the aforementioned two items.

#### Item Parameters

In the remaining items, only one item’s discrimination parameter of GRM is below 0.5 and then was deleted from the item bank. This item is “I prefer shopping for brand name merchandise.”

#### Item Fit Testing

It was found that the *p*-value of *S-*χ*^2^* of two items was less than 0.01, which indicated that these two items did not fit GRM and were deleted thereafter. After these two items were deleted, 85 items remained in the item bank.

#### Differential Item Functioning

To ensure the fairness of the CAT, the DIF test was performed on three group variables: gender, whether the participant was an only child, and the region (urban or rural). Results indicate that the *R*^2^ change for all items was less than 0.02 for each group variable. That is, none of the items in the item bank had DIF for each group variable.

#### The Psychometric Characteristic of the Final Item Bank

After 51 items that did not conform to the psychometric properties under the framework of IRT were removed, the remaining 85 items from the final item bank of CAT-NP met the unidimensional assumption, local independence assumption, high item discrimination, acceptable item fit, and no DIF. The item parameter characteristics of the final item bank are shown in Table 3, and the information of items contents are reported in the online Supplementary Material.

**TABLE 3:** Some information of final item bank.

**Item no.**	**Item parameters under CTT**		**Discrimination under IRT**	**Threshold (difficulty) Parameters under IRT**				**Item fit**
	**Threshold (difficulty)**	**Discrimination**	***a***	***b1***	***b2***	***b3***	***b4***	***p-*value of *S-*χ*^2^***
1	0.15	0.30	0.818	2.436	–	–	–	0.937
2	0.24	0.29	0.705	1.784	–	–	–	0.934
3	0.13	0.30	0.986	2.265	–	–	–	0.212
4	0.10	0.32	1.249	2.187	–	–	–	0.072
5	0.14	0.37	1.158	1.899	–	–	–	0.244
6	0.37	0.38	0.900	0.721	–	–	–	0.149
7	0.15	0.38	1.199	1.818	–	–	–	0.669
8	0.21	0.41	1.156	1.453	–	–	–	0.298
9	0.16	0.41	1.325	1.660	–	–	–	0.012
10	0.24	0.34	0.871	1.538	–	–	–	0.307
11	0.13	0.44	1.634	1.676	–	–	–	0.212
12	0.12	0.44	1.706	1.676	–	–	–	0.118
13	0.12	0.48	2.020	1.538	–	–	–	0.871
14	0.16	0.33	0.942	2.077	–	–	–	0.542
15	0.50	0.32	0.704	−0.010	–	–	–	0.021
16	0.35	0.32	0.688	1.031	–	–	–	0.576
17	0.43	0.47	1.200	0.306	–	–	–	0.275
18	0.14	0.49	1.958	1.483	–	–	–	0.208
19	0.11	0.48	2.191	1.587	–	–	–	0.455
20	0.24	0.39	1.031	1.363	–	–	–	0.691
21	0.16	0.42	1.328	1.623	–	–	–	0.680
22	0.17	0.44	1.418	1.532	–	–	–	0.796
23	0.15	0.48	1.738	1.475	–	–	–	0.034
24	0.17	0.41	1.258	1.610	–	–	–	0.132
25	0.12	0.48	2.062	1.564	–	–	–	0.031
26	0.14	0.50	1.959	1.453	–	–	–	0.335
27	0.24	0.33	0.779	1.646	–	–	–	0.903
28	0.34	0.39	0.927	0.876	–	–	–	0.494
29	0.32	0.42	1.067	0.861	–	–	–	0.525
30	0.41	0.31	0.656	0.596	–	–	–	0.207
31	0.39	0.42	1.003	0.523	–	–	–	0.282
32	0.11	0.47	2.146	1.599	–	–	–	0.104
33	0.25	0.36	0.885	1.425	–	–	–	0.908
34	0.23	0.51	1.618	1.075	–	–	–	0.591
35	0.20	0.43	1.313	1.408	–	–	–	0.367
36	0.24	0.41	1.123	1.305	–	–	–	0.807
37	0.15	0.56	2.438	1.284	–	–	–	0.323
38	0.11	0.50	2.257	1.557	–	–	–	0.867
39	0.12	0.48	1.946	1.581	–	–	–	0.131
40	0.24	0.49	1.422	1.118	–	–	–	0.173
41	0.26	0.43	1.184	1.145	–	–	–	0.991
42	0.39	0.35	0.799	0.651	–	–	–	0.617
43	0.18	0.45	1.477	1.386	–	–	–	0.576
44	0.19	0.44	1.378	1.404	–	–	–	0.320
45	0.54	0.30	0.667	−0.246	–	–	–	0.186
46	0.40	0.30	0.642	0.726	–	–	–	0.436
47	0.15	0.48	1.753	1.463	–	–	–	0.138
48	0.22	0.36	0.914	1.652	–	–	–	0.291
49	0.12	0.49	2.170	1.535	–	–	–	0.421
50	0.40	0.36	0.809	0.564	–	–	–	0.737
51	0.17	0.50	1.755	1.338	–	–	–	0.175
52	0.09	0.46	2.385	1.677	–	–	–	0.075
53	0.21	0.45	1.369	1.318	–	–	–	0.413
54	0.13	0.50	2.070	1.505	–	–	–	0.555
55	0.24	0.50	1.491	1.076	–	–	–	0.682
56	0.45	0.36	0.843	0.308	–	–	–	0.104
57	0.16	0.47	1.595	1.451	–	–	–	0.117
58	0.24	0.44	1.244	1.207	–	–	–	0.527
59	0.36	0.34	0.784	0.855	–	–	–	0.945
60	0.15	0.42	1.441	1.588	–	–	–	0.324
61	0.28	0.45	1.225	1.018	–	–	–	0.319
62	0.18	0.48	1.626	1.370	–	–	–	0.357
63	0.12	0.48	2.016	1.558	–	–	–	0.325
64	0.37	0.33	0.701	0.873	–	–	–	0.354
65	0.28	0.47	1.305	0.986	–	–	–	0.015
66	0.16	0.28	0.797	2.304	–	–	–	0.355
67	0.17	0.46	1.532	1.463	–	–	–	0.908
68	0.11	0.43	1.733	1.728	–	–	–	0.212
69	0.38	0.50	0.954	−2.318	−0.611	0.708	3.266	0.457
70	0.43	0.46	0.926	−3.188	−1.033	0.419	2.946	0.037
71	0.24	0.65	1.613	−0.659	0.595	1.400	2.542	0.477
72	0.26	0.67	1.715	−1.091	0.327	1.731	2.929	0.088
73	0.33	0.42	0.820	−2.772	−0.028	1.859	4.449	0.157
74	0.39	0.51	1.072	−2.121	−0.471	0.597	2.591	0.162
75	0.35	0.56	1.296	−2.093	−0.272	1.160	2.908	0.112
76	0.26	0.67	1.752	−0.900	0.572	1.362	2.759	0.175
77	0.27	0.60	1.416	−1.000	0.427	1.570	2.973	0.327
78	0.28	0.49	0.939	−1.967	0.639	2.141	4.122	0.584
79	0.41	0.37	0.700	−5.449	−2.013	1.551	5.146	0.688
80	0.34	0.50	1.005	−2.539	−0.113	1.484	3.624	0.524
81	0.24	0.46	0.868	−1.364	1.105	2.289	4.457	0.195
82	0.41	0.42	0.813	−3.704	−0.876	0.632	4.102	0.192
83	0.24	0.71	2.021	−0.919	0.673	1.511	2.688	0.670
84	0.29	0.67	1.743	−1.270	0.308	1.300	2.614	0.697
85	0.46	0.34	0.611	−5.404	−2.077	0.015	3.964	0.017

*a is the discrimination parameter; b is the threshold; the last 17 items are scored with 0, 1, 2, 3, 4, and the other items are scored with 0, 1.*

In Table 3, the discrimination parameters under IRT of items range from 0.61 to 2.44 with a mean of 1.315 (*SD* = 0.484), which shows that the final item bank has high discrimination and can discriminate different subjects with different trait levels of NP.

**FIGURE 1:**
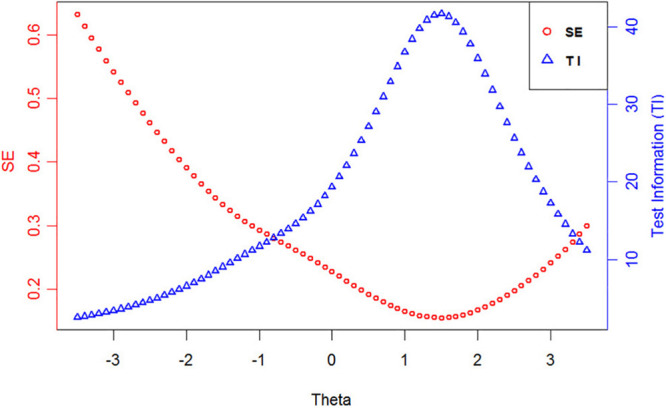
The test information and standard errors of the final item bank.

Test information can reflect the quality of the test in IRT analysis, and the square root of the test information is inversely proportional to the standard errors (*SE*) of measurement. The test information and standard errors of the final item bank are displayed in Figure 1. According to the equation (4), the reliability of the test reaches 0.9 when the test information is 10. Figure 1 shows that the reliability of the whole item bank is good, especially for subjects with a trait level between -1 and 3. In addition, the final item bank has a Cronbach’s alpha of 0.941.

### Simulation of the CAT-NP

#### Characteristics of the CAT-NP

Under different conditions of reliability, the results of CAT analysis based on real data are shown in Table 4, including the number of items used, mean *SE*, marginal reliability, and correlation between CAT θ and complete test θ.

**TABLE 4:** Characteristics of the CAT under several stopping rules.

**Stopping rule**	**Number of items used**			**Mean *SE* (θ)**	**Marginal reliability**	**Correlation^b^**	**Correlation with NPQ**
	**Mean**	***SD***	**% all^a^**				
None^c^	85	0	100%	0.227	0.945^d^	1.000	0.911
ρ ≥ 0.90	24.00	9.07	29%	0.322	0.895	0.966	0.833
ρ ≥ 0.85	12.88	6.37	15%	0.382	0.853	0.930	0.761
ρ ≥ 0.80	8.27	4.52	10%	0.434	0.811	0.906	0.724
ρ ≥ 0.75	6.44	3.67	8%	0.478	0.771	0.888	0.706
ρ ≥ 0.70	5.25	2.54	6%	0.520	0.729	0.874	0.693

*^*a*^The percentage of the mean number of items used in the full-item bank. ^*b*^Correlation between CAT θ and complete test θ. ^*c*^The entire item bank was administered. ^*d*^Coefficient alpha for the full test was 0.94.*

As the reliability decreased from 0.90 to 0.70, the average number of items required decreased from 24 to 5.25; that is, the percentage of an average number of items required in the total length of the item bank fell from 29 to 6%. The results show that the CAT can significantly save the test length and improve the test efficiency under those reliability levels, and the effect is more obvious when the precision requirement is lower.

**FIGURE 2:**
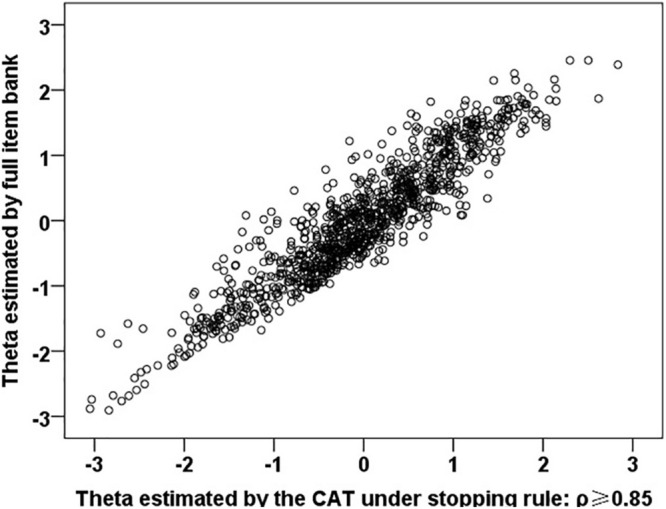
Correlation between the latent trait (θ) estimated by CAT (ρ ≥ 0.85) and full item bank.

When the reliability decreased from 0.9 to 0.7, the Pearson correlation coefficients between the latent trait estimate of CAT and the latent trait estimate of the full item bank decreased from 0.966 (*p* < 0.001) to 0.874 (*p* < 0.001). In particular, when ρ ≥ 0.90 and ρ ≥ 0.85, the average number of items required for CAT is only about 24 and 13 respectively, and the Pearson correlation coefficients are both greater than 0.9 (*p* < 0.001). The correlation scatter diagram under *ρ*≥ 0.85 is shown in Figure 2. In addition, the last column of Table 4 shows the correlation between CAT estimation and NPQ (73 items) estimation under these stopping rules, and there are significant moderate or high correlations.

**FIGURE 3:**
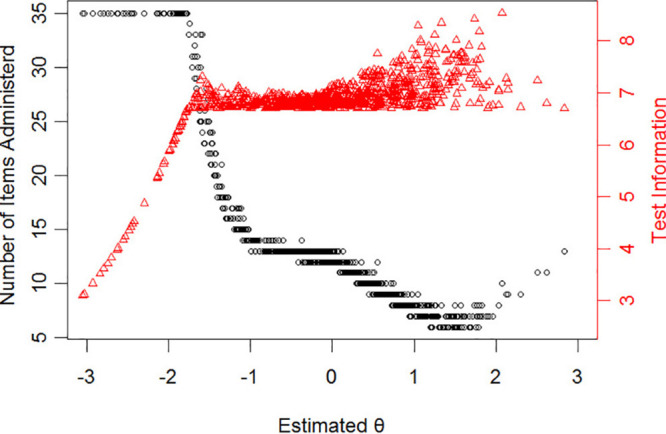
The relationship between the latent trait (θ) estimate and the number of administered items for stopping rule “ρ ≥ 0.85” (dots represent respondents). The trigon represents test information.

Figure 3 displays the number of selected items and test information for subjects with different levels of NP when the stopping rule is ρ ≥ 0.85. In Figure 3, with the increase of NP level, the number of items needed for accurate assessment of the participants showed a tendency to decline and then rise slightly. In particular, there were 936 participants (92% of total participants) whose NP level was higher than -1.5 estimated by CAT-NP (ρ ≥ 0.85) and the average number of items needed for an accurate assessment is 11 with test reliability above 0.85. As we can see from Table 4, when reliability is decreased from 0.9 to 0.85, the average number of items used in the test is reduced by more than half, and the correlation is not too low (*r* > 0.9). Therefore, with higher test efficiency, the accuracy of estimating the latent trait level of the subjects will be reduced. Hence, in practical application, the level of reliability should be selected according to the actual requirements.

**FIGURE 4:**
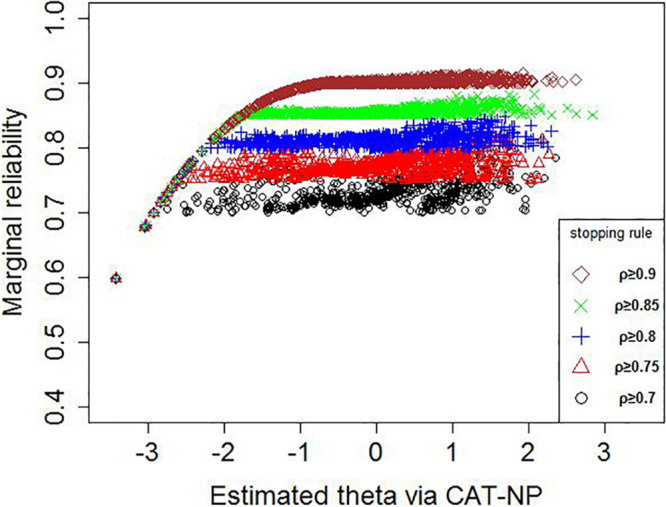
Reliability of 1,013 participants under several stopping rules.

Figure 4 shows the reliability of 1,013 participants under several stopping rules, and the reliability of all participants in the CAT-NP test was greater than 0.6. Overall, the CAT reliability of all participants with a theta greater than -1.5 can reach the criteria of the corresponding stopping rule.

#### Criterion-Related Validity and Predictive Utility (Sensitivity and Specificity) of CAT-NP

In this study, the measuring results of PDQ-4-NP scale were used as the criterion, and the criterion-related validity of CAT-NP is calculated (see Table 5). In addition, other results are shown in Table 5.

**TABLE 5:** The predictive utility (sensitivity and specificity) of CAT-NP under different stopping rules and the criterion-related validity.

**Stopping Rule**	**PDQ-4-NP**					
	**AUC (95%CI)**	**Cut-off**	**Se**	**Sp**	**YI**	**Criterion-related validity**
None	0.902 (0.877–0.927)	0.580	0.879	0.791	0.670	0.687***
ρ ≥ 0.90	0.880 (0.853–0.907)	0.418	0.912	0.723	0.635	0.632***
ρ ≥ 0.85	0.860 (0.829–0.891)	0.363	0.901	0.693	0.594	0.584***
ρ ≥ 0.80	0.852 (0.819–0.886)	0.680	0.747	0.815	0.562	0.561***
ρ ≥ 0.75	0.840 (0.805–0.876)	0.360	0.857	0.700	0.557	0.544***
ρ ≥ 0.70	0.838 (0.803–0.873)	0.025	0.923	0.582	0.505	0.541***

None, the entire item bank was administered; 95% CI, 95% confidence intervals; AUC, area under curve; Se, sensitivity; Sp, specificity; YI, Youden Index. ***p < 0.001.

Table 5 shows that the AUC in each stopping rule is greater than 0.8, which indicates that the diagnosis of CAT-NP is acceptable. Especially when reliability is not less than 0.9, the value of AUC is 0.880, which is close to the standard of 0.9. On the other hand, Table 5 shows that there is a significant moderate or high correlation (*p* < 0.001) between the estimated level of NP and the score of PDQ-4-NP scale under all the stopping rules mentioned earlier, which indicates that CAT-NP has acceptable convergent validity with PDQ-4-NP scale.

In sum, the constructed CAT-NP has acceptable reliability and validity for the real participants, and the efficiency of the test is high.

## Discussion

Research on CAT-NP is conducted to realize more efficient measurements of NP. Under the framework of IRT, this study first sets up an item bank (85 items) of NP, which meets with the psychometric properties of IRT. Then, based on the item bank, the simulation of CAT-NP is conducted to investigate the characteristics, marginal reliability, validity, and the predictive utility (sensitivity and specificity) of CAT-NP.

As far as the final items in CAT-NP, it can be seen from Table 4 and Figure 3 that the CAT-NP is more suitable for the measurement of individuals with severe NP, indicating that the item bank lacks some better-quality items for the low level of NP. There are 85 items in the final item bank of CAT-NP, although the average value of item discrimination is 1.31 (*SD* = 0.48), which indicates that the item has good quality. However, if we consider the issue of item exposure, the existing items can be further expanded. It is generally considered that the appropriate item bank size should be 6–12 times the number of paper-and-pencil test items (Stocking and Swanson, 1993). Future research needs to supplement the item bank with high-quality items and consider the issue of exposure control.

Regarding the issue of test reliability, it was pointed out that if we only want to compare whether there is a significant difference in test scores between two groups, then the reliability should only be 0.6–0.8. However, if the test is used to compare whether there is a difference in test scores between individuals, then the reliability should be at least 0.85 (Dai, 2015). The results of this study show that the CAT-NP could provide reasonable reliability and validity by answering 13 items on average in CAT-NP (ρ ≥ 0.85, *r* = 0.930, saved 85% items). Moreover, the value of AUC was close to 0.9, which indicates that CAT-NP had better measuring accuracy. In other words, if CAT-NP is applied in practice, it is recommended that the stopping rule is set to ρ ≥ 0.85. The most significant advantage of CAT is to minimize the number of item usage without losing measurement accuracy. Under the stopping rule given by CAT-NP, the average number of items is at least 70% less than that of the paper-and-pencil test. It is meaningful to study the CAT version of NP, which reduces the burden of testing. Also, each subject only does the items suitable for themselves from the large item bank, making the test more reliable and intelligent.

Because the CAT-NP in this study is a simulation study, there is an underlying assumption. The assumption is that the responses to the paper-and-pencil test were the same as those in the CAT test. Fortunately, it has been confirmed in many studies: the results of CAT reported by Potosky and Bobko (1997) are highly correlated with the traditional paper-and-pencil tests, and other studies also showed that the two test scenarios had no significant impact on the results. Moreover, in terms of the participants, the proportion of men and women participants in this study was not balanced enough, and the sampling was not broad enough. Also, it is difficult to ensure that the participants are always completing the 145 items seriously. These problems will affect the accuracy of CAT construction, which needs to be improved in future research. By improving the aforementioned shortcomings, a CAT-NP software will be built, which can greatly improve the use of this new measurement tool by clinicians or researchers.

In summary, the purpose of the CAT is to ensure the accuracy of the test and improve the efficiency of the test (Smits et al., 2011). This study aims to develop a CAT version of NP and in the process has also proved that CAT-NP can accurately and efficiently assess the level of NP. At present, many psychological tests use a paper-and-pencil test. Therefore, the application of CAT to more psychological tests should be addressed in future research. Many of the psychological tests are multidimensional scales, so it is worth noting that multidimensional scales may be a trend in practical applications. The authors of this article believe that multidimensional computerized adaptive testing (MCAT) or cognitive diagnostic computerized adaptive testing (CD-CAT) can be used to deal with multidimensional scales. With the development of CAT technology, its application will be continuously updated.

## Data Availability Statement

The datasets generated for this study are available on request to the corresponding author.

## Ethics Statement

The studies involving human participants were reviewed and approved by Ethics Committee of Center for Mental Health Education and Research of Jiangxi Normal University. The patients/participants provided their written informed consent to participate in this study.

## Author Contributions

HL analyzed and interpreted the data, and drafted the original manuscript. DT and YC assisted with the literature review and contributed to the manuscript’s revision. All authors approved the final manuscript for publication.

## Conflict of Interest

The authors declare that the research was conducted in the absence of any commercial or financial relationships that could be construed as a potential conflict of interest.

## References

[B1] Ab Saleh M. Awada A. (2016). A logical model for narcissistic personality disorder. *Int. J. Synth. Emot.* 7 69–87. 10.4018/IJSE.2016010106

[B2] Aiken L. R. (1997). *Psychological Testing and Assessment*, 9th Edn Washington, DC: American Psychological Association.

[B3] Akaike H. (1974). “IEEE xplore abstract - a new look at the statistical model identification,” in *Proceedings of the Automatic Control IEEE Transactions* (Piscataway, NJ: IEEE).

[B4] American Psychiatric Association (2013). *Diagnostic and Statistical Manual of Mental Disorders*, 5th Edn Washington, DC: Author.

[B5] Ames D. R. Rose P. Anderson C. P. (2006). The npi-16 as a short measure of narcissism. *J. Res. Pers.* 40 440–450. 10.1016/j.jrp.2005.03.002

[B6] Chalmers R. P. (2012). mirt: a multidimensional item response theory package for the R environment. *J. Stat. Softw.* 48 1–29. 10.18637/jss.v048.i06

[B7] Chang H. H. Ying Z. (1996). A global information approach to computerized adaptive testing. *Appl. Psychol. Meas.* 20 213–229. 10.1177/014662169602000303

[B8] Choi S. W. (2015). *Lordif: Logistic Ordinal Regression Differential Item Functioning using IRT.* Oxford: Oxford University Press.

[B9] Choi S. W. Grady M. W. Dodd B. G. (2011). A new stopping rule for computerized adaptive testing. *Educ. Psychol. Meas.* 71 37–53. 10.1177/0013164410387338 21278821 PMC3028267

[B10] Cohen J. (1988). *Statistical Power Analysis for the Behavioral Sciences.* Abingdon: Routledge.

[B11] Crane P. K. Gibbons L. E. Jolley L. Van B. G. (2006). Differential item functioning analysis with ordinal logistic regression techniques. *Med. Care* 44 115–123. 10.1097/01.mlr.0000245183.28384.ed 17060818

[B12] Crockatt P. (2006). Freud’s ‘On narcissism: an introduction’. *J. Child Psychother.* 32 4–20.

[B13] Dai H.-Q. (2015). *Psychometrics.* Beijing, BJ: Higher Education Press.

[B14] Drasgow F. Olson-Buchanan J. (eds) (1999). *Innovations in Computerized Assessment.* New York, NY: Psychology Press.

[B15] Egberink I. J. Veldkamp B. P. (2007). “The development of a computerized adaptive test for integrity,” in *Proceedings of the 2007 GMAC Conference on Computerized Adaptive Testing*, Minneapolis, MN.

[B16] Embretson S. E. Reise S. P. (2000). *Multivariate Applications Books Series. Item Response Theory for Psychologists.* Mahwah, NJ: Lawrence Erlbaum Associates Publishers.

[B17] First M. B. Spitzer R. L. Gibbon M. Williams G. B. W. Benjamin L. (1995). *Structural Clinical Interview for DSM-IV Axis II Personality Disorders (SCID-II).* Washington, DC: American Psychiatric Press.

[B18] Flens G. Smits N. Carlier I. van Hemert A. M. de Beurs E. (2016). Simulating computer adaptive testing with the mood and anxiety symptom questionnaire. *Psychol. Assess.* 28 953. 10.1037/pas0000240 26691506

[B19] Fliege H. Becker J. Walter O. B. Bjorner J. B. Rose K. M. (2005). Development of a computer-adaptive test for depression (d-cat). *Qual. Life Res.* 14 2277–2291. 10.2307/4039962 16328907

[B20] Forkmann T. Kroehne U. Wirtz M. Norra C. Baumeister H. Gauggel S. (2013). Adaptive measuring for depression - Recalibration of an item bank for the assessment of depression in persons with mental and somatic diseases and evaluation in a simulated computer-adaptive test environment. *J. Psychos. Res.* 75 437–443. 10.1016/j.jpsychores.2013.08.022 24182632

[B21] Frederic P. Miller A. F. V. John M. (2010). *History of Narcissism.* Mishawaka: Alphascript Publishing.

[B22] Furnham A. Milner R. Akhtar R. Fruyt F. D. (2014). A review of the measures designed to assess dsm-5 personality disorders. *Psychology* 5 1646–1686. 10.4236/psych.2014.514175

[B23] Gaynes B. N. Burns B. J. Tweed D. L. Erickson P. (2002). Depression and health-related quality of life. *J. Nervous Ment. Dis.* 190 799–806. 10.1097/01.NMD.0000041956.05334.07 12486367

[B24] Gibbons R. D. Weiss D. J. Pilkonis P. A. Frank E. Moore T. Kim J. B. (2014). Development of the cat-anx: a computerized adaptive test for anxiety. *Am. J. Psychiatry* 171 187–194. 10.1176/appi.ajp.2013.13020178 23929270 PMC4052830

[B25] Glover N. Miller J. D. Lynam D. R. Crego C. Widiger T. A. (2012). The five-factor narcissism inventory: a five-factor measure of narcissistic personality traits. *J. Pers. Assess.* 94 500–512. 10.1080/00223891.2012.670680 22475323

[B26] Goldberg L. R. Johnson J. A. Eber H. W. Hogan R. Ashton M. C. Cloninger C. R. (2006). The international personality item pool and the future of public-domain personality measures. *J. Res. Pers.* 40 84–96. 10.1016/j.jrp.2005.08.007

[B27] Hyler S. E. (1994). *PDQ-4 and PDQ-4+ Instructions for Use.* New York, NY: New York State Psychiatric Institute.

[B28] Kang T. Chen T. T. (2008). Performance of the generalized S-X2 item fit index for polytomous IRT models. *J. Educ. Meas.* 45 391–406. 10.2307/20461906

[B29] Kraemer H. C. Kupfer D. J. (2006). Size of treatment effects and their importance to clinical research and practice. *Biol. Psychiatry* 59 990–996. 10.1016/j.biopsych.2005.09.014 16368078

[B30] Masters G. N. Wright B. D. (1997). *The Partial Credit Model. In Handbook of Modern Item Response Theory.* New York, NY: Springer, 101–121.

[B31] Miller J. D. Campbell W. K. (2008). Comparing clinical and social-personality conceptualizations of narcissism. *J. Pers.* 76 449–476. 10.1111/j.1467-6494.2008.00492.x 18399956

[B32] Moore T. M. Calkins M. E. Reise S. P. Gur R. C. Gur R. E. (2018). Development and public release of a computerized adaptive (cat) version of the schizotypal personality questionnaire. *Psychiatry Res.* 263 250–256. 10.1016/j.psychres.2018.02.022 29625786 PMC5911247

[B33] Motter E. H. (2009). *Preliminary Study of the Narcissistic Personality Questionnaire.* Bachelor dissertation, Mount Vernon Nazarene University, Mount Vernon, MV.

[B34] Muraki E. (1997). *A Generalized Partial Credit Model. Handbook of Modern Item Response Theory.* New York, NY: Springer.

[B35] Orlando M. Thissen D. (2003). Further investigation of the performance of S-X2: an item fit index for use with dichotomous item response theory models. *Appl. Psychol. Meas.* 27 289–298. 10.1177/0146621603027004004

[B36] Pilkonis P. A. Yu L. Dodds N. E. Johnston K. L. Maihoefer C. C. Lawrence S. M. (2014). Validation of the depression item bank from the patient-reported outcomes measurement information system (promis) in a three-month observational study. *J. Psychiatr. Res.* 56 112–119. 10.1016/j.jpsychires.2014.05.010 24931848 PMC4096965

[B37] Potosky D. Bobko P. (1997). Computer versus paper-and-pencil administration mode and response distortion in noncognitive selection tests. *J. Appl. Psychol.* 82 293–299. 10.1037/0021-9010.82.2.293 9109287

[B38] Raine A. (1991). The spq: a scale for the assessment of schizotypal personality based on dsm-III-r criteria. *Schizophr. Bull.* 17 555–564. 10.1093/schbul/17.4.555 1805349

[B39] Reckase M. D. (1979). Unifactor latent trait models applied to multifactor tests: results and implications. *J. Educ. Stat.* 4 207–230. 10.3102/10769986004003207

[B40] Reise S. P. Henson J. M. (2000). Computerization and adaptive administration of the NEO PI-R. *Assessment* 7 347–364. 10.1016/0043-1354(74)90109-2 11151961

[B41] Samejima F. (1969). Estimation of latent ability using a response pattern of graded scores. *Psychometrika* 34 1–97. 10.1007/BF03372160

[B42] Schisterman E. F. Perkins N. J. Liu A. Bondell H. (2005). Optimal cut-point and its corresponding youden index to discriminate individuals using pooled blood samples. *Epidemiology* 16 73–81. 10.1097/01.ede.0000147512.81966.ba 15613948

[B43] Schwarz G. (1978). Estimating the dimension of a model. *Ann. Stat.* 6 461–464. 10.1214/aos/1176344136

[B44] Simms L. J. Goldberg L. R. Roberts J. E. Watson D. Welte J. Rotterman J. H. (2011). Computerized adaptive assessment of personality disorder: introducing the cat–pd project. *J. Pers. Assess.* 93 380–389. 10.1080/00223891.2011.577475 22804677 PMC3400119

[B45] Smits N. Cuijpers P. Straten A. V. (2011). Applying computerized adaptive testing to the ces-d scale: a simulation study. *Psychiatry Res.* 188 147–155. 10.1016/j.psychres.2010.12.001 21208660

[B46] Spiegelhalter D. Best N. Carlin B. der Linde A. V. (1998). Bayesian deviance, the effective number of parameters, and the comparison of arbitrarily complex models. *Res. Rep.* 64 98–99.

[B47] Stocking M. L. Swanson L. (1993). A method for severely constrained item selection in adaptive testing. *Appl. Psychol. Meas.* 17 277–292. 10.1002/j.2333-8504.1992.tb01468.x

[B48] Twenge J. M. Miller J. D. Campbell W. K. (2014). The narcissism epidemic: commentary on modernity and narcissistic personality disorder. *Pers. Disord. Theory Res. Treat.* 5 227–229. 10.1037/per0000008 24796568

[B49] Wainer H. Dorans N. J. Flaugher R. Green B. F. Mislevy R. J. (2000). *Computerized Adaptive Testing: A Primer.* Abingdon: Routledge.

[B50] Wainer H. Dorans N. J. Green B. F. Steinberg L. Flaugher R. Mislevy R. J. (2001). Computerized adaptive testing: a primer. *Qual. Life Res.* 10 733–734. 10.1023/A:1016834001219

[B51] Weiss D. J. Kingsbury G. G. (1984). Application of computerized adaptive testing to educational problems. *J. Educ. Meas.* 21 361–375. 10.1111/j.1745-3984.1984.tb01040.x

[B52] Yang J. Mccrae R. R. Costa P. T. Yao S. Dai X. Cai T. (2000). The cross-cultural generalizability of axis-ii constructs: an evaluation of two personality disorder assessment instruments in the People’s Republic of China. *J. Pers. Disord.* 14 249–263. 10.1521/pedi.2000.14.3.249 11019748

[B53] Yen W. M. (1993). Scaling performance assessments: strategies for managing local item dependence. *J. Educ. Meas.* 30 187–213. 10.1111/j.1745-3984.1993.tb00423.x

