# Supplementary material for: Procedures to Develop a Computerized Adaptive Testing to Advance the Measurement of Narcissistic Personality
Source: Front Psychol. 2020 Jun 30;11:1437. doi: 10.3389/fpsyg.2020.01437 (PMC7344143; doi:10.3389/fpsyg.2020.01437)
Supplement: Supplementary file 1 [file Table_1.DOC]

**Table .** The item bank of the final CAT-NP.

| Original  no. | Item score | | Item contents |
| --- | --- | --- | --- |
|  | M±SD | Grade |  |
| 1 | 0.15±0.35 | 2 | 1. I know that I am good because everybody keeps telling me so. |
| 12 | 0.24±0.43 | 2 | 1. People always seem to recognize my authority. |
| 13 | 0.13±0.34 | 2 | 1. I am going to be a great person. |
| 15 | 0.10±0.30 | 2 | 1. I am more capable than other people. |
| 77 | 0.14±0.35 | 2 | 1. When you have a problem, do you almost always insist on meeting with senior leaders? |
| 79 | 0.37±0.48 | 2 | 1. Is it important for you that people notice or admire you in some way? |
| 81 | 0.15±0.36 | 2 | 1. You often think that others should give you special treatment, is it? |
| 82 | 0.21±0.41 | 2 | 1. Do you often think it's necessary to offend people in order to get what you want? |
| 83 | 0.16±0.36 | 2 | 1. Do you often have to put your needs above others? |
| 84 | 0.24±0.43 | 2 | 1. Do you often hope that others will respect you and do what you want without a doubt? |
| 85 | 0.13±0.33 | 2 | 1. Do you often think that it doesn't matter how you treat others' thoughts or feelings? |
| 86 | 0.12±0.33 | 2 | 1. Do you get angry when others do a good job? |
| 87 | 0.12±0.33 | 2 | 1. Do you think others are always envy you? |
| 88 | 0.16±0.36 | 2 | 1. Do you think there are few people worth your time? |
| 18 | 0.50±0.50 | 2 | 1. I often dream that I will be a great person. |
| 44 | 0.35±0.48 | 2 | 1. I really need someone to pay attention or praise me. |
| 1 | 0.43±0.50 | 2 | 1. Many of my ideas are ingenious but people have difficulty in understanding how great they are. |
| 3 | 0.14±034 | 2 | 1. I often try to fly first class so that I can meet people like myself. |
| 5 | 0.11±0.31 | 2 | 1. When I go to restaurants I try to get special service. |
| 7 | 0.24±0.43 | 2 | 1. I don’t have much patience to listen to other people’s problems. |
| 9 | 0.16±0.37 | 2 | 1. People have told me that I am patronizing. |
| 11 | 0.17±0.37 | 2 | 1. I have had several brilliant business schemes. |
| 12 | 0.15±0.36 | 2 | 1. People who do well in my line of work don’t associate with unsuccessful people. |
| 14 | 0.17±0.38 | 2 | 1. I have no trouble in complaining about poor service. |
| 17 | 0.12±0.32 | 2 | 1. The success of some of the people I know makes me resent them. |
| 19 | 0.14±0.35 | 2 | 1. Very few people in my line of work have my skills. |
| 20 | 0.24±0.43 | 2 | 1. I have had people tell me that I look like a movie star. |
| 21 | 0.34±0.47 | 2 | 1. I often find that I can only be understood by people like myself. |
| 22 | 0.32±0.47 | 2 | 1. I don’t get enough praise for my ideas or work. |
| 26 | 0.41±0.4 | 2 | 1. I sometimes envy people who have more money than me. |
| 29 | 0.39±0.49 | 2 | 1. I wish I was more famous. |
| 30 | 0.11±0.31 | 2 | 1. I seldom (rarely) like to associate with ordinary people. |
| 31 | 0.25±0.43 | 2 | 1. People who know of my talents don’t hesitate to contact me. |
| 33 | 0.23±0.42 | 2 | 1. I often get people to do what I want. |
| 35 | 0.20±0.40 | 2 | 1. People sometimes appear to be envious of my achievements. |
| 37 | 0.24±0.42 | 2 | 1. People often admire my accomplishments. |
| 41 | 0.15±0.36 | 2 | 1. I have a knack for getting special treatment. |
| 42 | 0.11±0.31 | 2 | 1. I have taken advantage of someone because it was the only way to get what I wanted. |
| 44 | 0.12±0.33 | 2 | 1. The attitude of some people who have “made it” makes me angry. |
| 45 | 0.24±0.43 | 2 | 1. People who don’t have my skills shouldn’t bother to be in my profession. |
| 50 | 0.26±0.44 | 2 | 1. I have a reputation for having others do whatever I say. |
| 51 | 0.39±0.49 | 2 | 1. Sometimes, when there is no other option, it is legitimate to take advantage of other people. |
| 53 | 0.18±0.39 | 2 | 1. Sometimes I feel that people envy my unique abilities. |
| 55 | 0.19±0.39 | 2 | 1. Sometimes I feel that people are unwilling to admit to how successful I am. |
| 59 | 0.54±0.50 | 2 | 1. People like me should be paid more for their hard work. |
| 60 | 0.40±0.49 | 2 | 1. People often manipulate others for their own needs. |
| 64 | 0.15±0.36 | 2 | 1. I find it hard to find people who are as creative as I am. |
| 70 | 0.22±0.41 | 2 | 1. It bores me to listen to other people’s sob stories. |
| 71 | 0.12±0.32 | 2 | 1. There have been times when people have ended relationships with me because they were envious of my talents. |
| 75 | 0.40±0.49 | 2 | 1. People who don’t come from my background rarely understand me. |
| 76 | 0.17±0.38 | 2 | 1. My accomplishments are truly unique but are not recognized as much as I would like. |
| 77 | 0.09±0.29 | 2 | 1. I am often bumped up to first class. |
| 79 | 0.21±0.40 | 2 | 1. When I read about the accomplishments of people I know in the newspaper, I often know that the reporters are exaggerating their contributions. |
| 81 | 0.13±0.33 | 2 | 1. Most of the people in my work place don’t have my kills. |
| 82 | 0.24±0.43 | 2 | 1. I often wonder what it would be like to be so famous that people will recognize me on the street. |
| 83 | 0.45±0.50 | 2 | 1. I prefer befriending people who are important. |
| 90 | 0.16±0.37 | 2 | 1. I know a lot of important and successful people. |
| 91 | 0.24±0.43 | 2 | 1. When I am in a social gathering, I enjoy being the center of attention. |
| 95 | 0.36±0.48 | 2 | 1. It is not nice to talk down to people but sometimes I don’t have any other choice. |
| 97 | 0.15±0.36 | 2 | 1. I am very skilled at getting people to do what I want. |
| 99 | 0.28±0.45 | 2 | 1. I am very aware of whether people have attended a good school/college. |
| 4 | 0.18±0.38 | 2 | 1. People enjoy hearing about my accomplishments. |
| 74 | 0.12±0.33 | 2 | 1. I have better taste in clothes than most people. |
| 6 | 0.37±0.48 | 2 | 1. If someone develops somebody else’s idea they should get the credit. |
| 32 | 0.28±0.45 | 2 | 1. I get irritated when I don’t get the treatment I deserve. |
| 80 | 0.16±0.37 | 2 | 1. When someone is successful he has every right to get special service. |
| 68 | 0.17±0.37 | 2 | 1. I often try to find ways to get special discounts that are not commonly available. |
| 61 | 0.11±0.32 | 2 | 1. When I see someone get hurt, I tend to remain calm. |
| 7 | 1.92±1.14 | 5 | 1. I often feel consumed with thoughts about great things I’m going to do. |
| 117 | 2.15±1.08 | 5 | 1. I am a superior person. |
| 19 | 1.22±1.15 | 5 | 1. I need positive attention from others to make me feel stronger. |
| 72 | 1.31±0.98 | 5 | 1. Other persons tend to be envious of me. |
| 27 | 1.63±1.00 | 5 | 1. I succeed at everything I try. |
| 52 | 1.96±1.19 | 5 | 1. I often fantasize about having lots of success and power. |
| 102 | 1.76±1.02 | 5 | 1. I do not waste my time hanging out with people who are beneath me. |
| 42 | 1.28±1.08 | 5 | 1. I only associate with people of my caliber. |
| 144 | 1.33±1.09 | 5 | 1. I’m not embarrassed to admit that I tend to use others. |
| 71 | 1.38±1.00 | 5 | 1. I don’t worry about others’ needs. |
| 132 | 2.06±0.81 | 5 | 1. Some people think I’m cocky or arrogant. |
| 116 | 1.69±1.01 | 5 | 1. I’m not big on feelings of sympathy. |
| 86 | 1.21±1.05 | 5 | 1. I don’t generally pay much attention to the woes of others. |
| 127 | 2.03±1.03 | 5 | 1. Someday I believe that most people will know my name. |
| 130 | 1.20±0.99 | 5 | 1. I believe I am entitled to special accommodations. |
| 55 | 1.44±1.05 | 5 | 1. I deserve to receive special treatment. |
| 49 | 2.32±1.02 | 5 | 1. It’s not a good day until someone notices something praiseworthy I’ve done. |

*Note:* The first column refers to the number of the item in the original scale. items 1~4 are from NPI-16, items 5~14 are from SCID-II, items 15~16 are from PDQ-4, items 17~68 are from NPQ, and items 69~85 are from FFNI.
